# The Use of Virtual Reality Technologies in the Treatment of Duchenne Muscular Dystrophy: Systematic Review

**DOI:** 10.2196/21576

**Published:** 2020-12-08

**Authors:** Maria Rosa Baeza-Barragán, Maria Teresa Labajos Manzanares, Carmen Ruiz Vergara, María Jesús Casuso-Holgado, Rocío Martín-Valero

**Affiliations:** 1 Department of Physiotherapy, Faculty of Health Sciences University of Malaga Malaga Spain; 2 Department of Physiotherapy University of Seville Seville Spain

**Keywords:** Duchenne muscular dystrophy, virtual reality, upper limb, physical therapy, muscular dystrophy, mutation, muscle, degeneration

## Abstract

**Background:**

Duchenne muscular dystrophy is a serious and progressive disease affecting one in 3500-6000 live male births. The use of new virtual reality technologies has revolutionized the world of youth rehabilitation.

**Objective:**

We performed a systematic review to study the effectiveness of the use of virtual reality systems applied in the rehabilitation of the upper limbs of individuals with Duchenne muscular dystrophy.

**Methods:**

Between June 2018 and September 2019, we carried out a series of searches in 5 scientific databases: (1) PubMed, (2) Web of Science, (3) Scopus, (4) The Cochrane Library, and (5) MEDLINE via EBSCO. Two evaluators independently conducted the searches following the PRISMA recommendations for systematic reviews for articles. Two independent evaluators collated the results. Article quality was determined using the PEDro scale.

**Results:**

A total of 7 clinical trials were included in the final review. These studies used new technologies as tools for physiotherapeutic rehabilitation of the upper limbs of patients with Duchenne muscular dystrophy. Collectively, the studies showed improvement in functionality, quality of life, and motivation with the use of virtual reality technologies in the rehabilitation of upper limbs of individuals with Duchenne muscular dystrophy.

**Conclusions:**

The treatment of neuromuscular diseases has changed in recent years, from palliative symptom management to preventive methods for capacity building. The use of virtual reality is beginning to be necessary in the treatment of progressive diseases involving movement difficulties, as it provides freedom and facilitates the improvement of results in capacity training. Given that new technologies are increasingly accessible, rehabilitation and physiotherapy programs can use these technologies more frequently, and virtual reality environments can be used to improve task performance, which is essential for people with disabilities. Ultimately, virtual reality can be a great tool for physiotherapy and can be used for Duchenne muscular dystrophy rehabilitation programs to improve patient performance during training.

**Trial Registration:**

PROSPERO International Prospective Register of Systematic Reviews CRD42018102548; https://www.crd.york.ac.uk/prospero/display_record.php?RecordID=102548

## Introduction

Duchenne muscular dystrophy is caused by a mutation in the gene that produces dystrophin, which is responsible for maintaining muscle properties. Duchenne muscular dystrophy is a rare disease with an incidence of 1 in 3500-6000 live male births [1]. The lack of dystrophin leads to a progressive degeneration of muscle fibers, which then become connective tissue and fat [2]. Currently, this disease has no cure. The main symptoms are muscle weakness, which progressively leads to a loss of function and independence, and, in advanced stages of the disease, a compromised respiratory system [3]. Functional tests are performed during medical assessments of children with Duchenne muscular dystrophy [4]. The Motor Function Measurement test is used to measure patients’ conditions before and after virtual task training [5]. Due to its analytical simplicity, the Vignos scale is also used to evaluate functionality and overall muscle performance in neuromuscular diseases. The Egen Scale Klassification was specially developed to measure the degree of functional impairment in daily living activities experienced by those with Duchenne muscular dystrophy [5].

Over time, there have been numerous guides for interprofessional action for affected individuals and their families [2]. Various technologies are used to provide patients and professionals with reliable forms of evaluation and effective treatments [4]. For instance, the use of virtual reality during treatment can provide a fun environment for patients [5,6]. Virtual reality may use multiple devices such as glasses, game consoles, immersion systems, applications for tablets and smartphones, gloves, exoskeletons, tele-rehabilitation systems, and more [7,8]. Virtual environments can involve representations of the users (ie, avatars), communication skills, the construction of or interaction with 3D objects, and the illusion of space [8]. From the combination of the concepts of virtual reality and rehabilitation, the concept of virtual rehabilitation has emerged, a term initially coined by Thalmann and Burdea [6]. Simulations are defined as learning contexts that attempt to imitate real-life situations [7]. Games should be designed to improve learning and promote autonomy [7].

The World Health Organization defines mHealth as the practice of medicine and public health supported by mobile devices such as mobile phones, patient monitoring devices, digital personal assistants, and other wireless devices [9]. Technology, like virtual reality, is used in health care settings to encourage therapists to adapt physical exercises in order to encourage patient participation [10]. New technologies enable the creation of virtual environments that capture patients’ attention by showing them interactive systems based on physical therapy exercises [11]. In this context, patients are continually challenged by constantly changing tasks, which elicit more active participation in the requested exercises and can potentially improve the intended results, thus accelerating the recovery process [6]. For example, training with commercial video games is used to help children with motor problems [12,13]. In recent years, virtual development has focused on robotic therapy (eg, the use of technology involving an exoskeleton) to improve distal movements in the hands [14,15].

Although no systematic review has been performed in children with Duchenne muscular dystrophy, a systematic review of the effectiveness of virtual reality in the manual function of children with cerebral palsy demonstrated little evidence of effectiveness [16]. Compared to those with cerebral palsy, patients with Duchenne muscular dystrophy retain musculature strength of the upper extremities for longer than they do in the lower extremities, especially in the distal muscles [17]. In those with cerebral palsy, distal muscles required for fine motor movements are the most affected in cases of hemiparesis and tetraparesis [16].

Virtual reality games in individuals with Duchenne muscular dystrophy who have considerable and progressive loss of movement could help to create safe rehabilitative environments in which to improve responsiveness or regulate treatment strategies [18]. The objective of this systematic review is to verify the effectiveness of virtual reality physiotherapy treatments in the rehabilitation of the upper limbs in individuals with Duchenne muscular dystrophy.

## Methods

### Search Strategy

**Figure 1:**
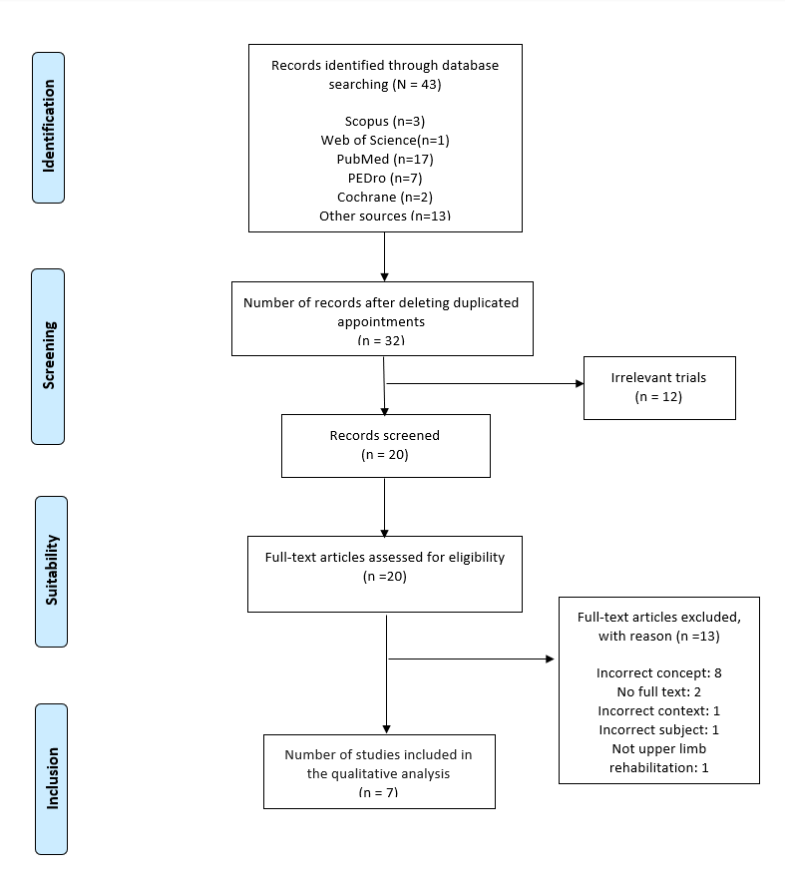
Literature search and study selection.

This systematic review is registered in PROSPERO with the code CRD42018102548. We used the most important evaluation items for systematic reviews and meta-analysis guides. We carried out a series of searches following PRISMA [19] recommendations for systematic reviews in 5 scientific databases: (1) PubMed, (2) Web of Science, (3) Scopus, (4) The Cochrane Library, and (5) MEDLINE via EBSCO. The searches were carried out between June 2018 and September 2019. Complete articles in English or Spanish were required. The following keywords from the Medical Subject Headings tree were used for the search: physical therapy, physiotherapy, upper limb, VR, new technologies, Duchenne, Duchenne muscular dystrophy, physical therapy modalities, virtual reality, and virtual reality exposure therapy. See Figure 1 for a complete list of the search strategy and terms used.

### Study Objective

The research question was: “Is the use of virtual reality in rehabilitation of the upper limbs effective in children with Duchenne muscular dystrophy?”

The following was derived using the PICO model [19]:

- P (patient): individuals with Duchenne muscular dystrophy.
- I (intervention): physical therapy with virtual reality.
- C (comparison): traditional physiotherapy.
- O (outcomes or results): effectiveness of use. Patient’s status.

### Eligibility Criteria

When searching the databases, a series of filters were chosen to limit the searches and select the articles. These were:

- Articles published between 2000 and 2019 inclusive (to ensure the relevance of the material).
- Articles whose subject of study were human.
- Randomized clinical trials.
- Articles written in English or Spanish.
- Studies based on physiotherapy applied with new technologies, including interventions with virtual reality, virtual games, and applications on tablets or smartphones.

### Data Extraction and Analysis

After searching different keywords in the aforementioned list of databases and sorting articles by title and summary, relevant articles were identified for complete reading, duplicate articles were eliminated, and the inclusion and exclusion criteria were applied to the sample of definitive data (Figure 1).

### Methodological Quality Assessment

We evaluated the methodological quality and internal validity of the studies using the PEDro scale. The PEDro scale (0-10) is based on the Delphi list developed by Verhagen et al [20]. Two independent evaluators (RMV and RBB) used the PEDro checklist to score each study. A study with a score of 4-5 was considered poor or acceptable, where a score below 4 was considered to indicate low methodological quality. Studies with a score below 6 were considered as having low or level 1 evidence, where a study with a score of 6-8 was considered good and a study with a score of 9-10 was considered excellent.

## Results

### Study Selection and PEDro Assessment

A total of 43 articles were identified for review. Duplicate articles were removed, leaving a total of 32 articles. Twelve of these were excluded as they were considered irrelevant. A total of 20 articles were screened and assessed for eligibility after reading the full text. Thirteen articles were excluded during the eligibility assessment phase: 8 because they showed an incorrect concept, 2 because they did not show the full text, 1 because it did not focus on Duchenne muscular dystrophy subjects, 1 due to incorrect context, and 1 because it did not display upper limb rehabilitation in the variables. The final complete quality assessment included 7 articles.

After deleting duplicates, 32 articles were identified through the keyword search, 20 of which were selected for screening and assessed for eligibility. After a full-text reading, only 7 were included in the qualitative analysis (Figure 1).

The 7 selected studies were published between 2009 and 2019. Of the 7 studies, 6 were carried out in Brazil and 1 in the Netherlands. Using the PEDro scale, 3 studies received a score of 7 and 1 received a score of 8. These studies were considered “good.” Two studies received a score of 6 and 1 study received a score of 5. These studies were considered “acceptable.” Criteria and scoring are depicted in Table 1.

**Table 1:** Methodological quality review of the included studies using the PEDro evaluation scale.

	Correa et al [18]	Capellini et al [5]	Heuntick et al [21]	Massetti et al [22]	Quadrado et al [23]	Malheiros et al [24]	De Freitas et al [17]
Eligibility criteria	✓	✓		✓	✓	✓	✓
Randomized	✓	✓	✓	✓	✓		✓
Allocation concealed	✓			✓	✓		
Baseline comparability		✓	✓	✓	✓	✓	✓
Subject blinding							
Therapist blinding							
Evaluator blinding			✓				
Adequate follow-up	✓	✓		✓	✓	✓	✓
Intention-to-treat analysis	✓	✓		✓	✓	✓	✓
Between-group comparisons	✓	✓	✓	✓	✓	✓	✓
Specific measurements and variability	✓	✓	✓	✓	✓	✓	✓
Total	7	7	5	8	7	6	6

### Assessment of Study Design

Collectively, the studies enrolled individuals with Duchenne muscular dystrophy aged 5 to 34 years. Individuals with Duchenne muscular dystrophy were ambulatory or wheelchair-dependent. Control groups included those with typical development. The number of individuals in the intervention groups varied. The largest included 60 participants [17], and the smallest included 19 [21]. Only 2 studies compared 2 groups of individuals with Duchenne muscular dystrophy during the intervention and control periods [21,22].

Total study length ranged from 1 day to 20 weeks. Heuntick et al [21] reported training with the virtual reality task for 20 weeks at home. Correa et al [18] reported 12 cumulative days of weekly intervention. Five studies measured the time it took participants to complete tasks they had been assigned in each of the following 3 phases: (1) acquisition, (2) retention, and (3) transfer [5,17,22-24]. These 5 studies agreed with the number of attempts in each phase, with attempts being higher in the acquisition phase (20-30 trials) than retention phase (5 trials) and transfer phase (5 trials).

The most commonly used variable was Motor Function Measurement, which measures limb functionality in 3 dimensions. The scores of Dimension 1 (D1: standing and transfer), Dimension 2 (D2: axial and proximal limb), and Dimension 3 (D3: distal limb) can predict functionality improvements [5,17,24]. However, some of the authors whose articles were selected for review do not believe that the Motor Function Measurement score is predictive of improvement, but instead believe that the time to complete the tasks assigned in each phase is predictive of improvement [22,23]. The relation between performance and Motor Function Measurement-D1 indicates that the use of virtual technology like smartphones is partly reliant on the muscles responsible for standing and transfer, as these muscles allow the head to look at the screen [5]. The dependent variable reported in studies to compare between phases is the movement time or time to perform [5,22-24]. Only one study included a motivation scale (Likert Scale) [18], and only one included a quality of life variable (Kidscreen-52) [21].

Virtual reality games can be simple, allowing individuals to adapt quickly and perform the tasks without problems [24]. Games used in the studies included in the review included musical games, virtual ball mazes, catching cubes, or labyrinths. Study participants used a variety of devices or virtual interfaces such as computers, webcams, touch screens, Kinect sensors, Leap Motion interfaces, simulated sounds, video consoles (like PlayStation II), and smartphones [5,17,18,21-24]. De Freitas et al [17] compared different interfaces with or without physical contact.

### Study Outcomes

Complete summaries of the included studies and their respective intervention details are presented in Table 2 and Table 3.

Study outcomes showed that the grade of motivation improved when using virtual devices [18]. Furthermore, a better performance was seen with a smartphone if a previous learning phase was used [5]. Improvements in the quality of life and elbow extension were seen if training with virtual reality games was performed at home [21].

Although Quadrado et al [23] found that conducting a timed task in a virtual environment facilitated real-life completion of that same task for individuals with Duchenne muscular dystrophy, Massetti et al [22] found no transference of learning between environments when comparing real-life and virtual tasks. Finally, individuals with Duchenne muscular dystrophy showed better performance when using interfaces without contact (like Leap Motion and Kinect) compared to touch screen interfaces [17]. No adverse effects of the use of virtual reality were described in the studies included in our review.

**Table 2:** Main characteristics of participants and results of the included studies.

Author, country	Population	Results	Statistical effect
Correa et al (2009) [18], Brazil	16 individuals with DMD^a^, 17-24 years old.	In satisfaction surveys, both patients and therapists found intervention with GenVirtual beneficial. Positive effects for patients with movement restriction.	The level of motivation using GenVirtual was greater (54%) compared to those not using the system (40%).
Capelini et al (2017) [5], Brazil	IG^b^=50 individuals with DMD; CG^c^=50 typically developing individuals. 10-34 years old.	IG showed better performance within a short-term motor learning protocol with smartphones. The score of MFM^d^ in standing position and transfers and the first attempt in the acquisition phase predicted the degree of learning.	Acquisition: IG=7.7-6.3 seconds; CG=4.9-4.1 seconds. Retention: IG=6.3 seconds; CG= 4.1 seconds. Transfer phase 2: IG retention=6.4 seconds; transfer phase 2=7.6 seconds.
Heuntinck et al (2018) [21], Brazil	IG=9 typically developing, ambulatory individuals; CG=10 wheelchair dependent individuals with DMD.	Elbow extension increased in the IG and decreased in the CG. The CG was not assisted by a coach, while the IG was. Elbow dimension=–0.6. Kidscreen showed a better quality of life.	IG SD=22.1 Dynamometer elbow extension: IG SD=1.8, *P*=.018; CG SD=1.6, *P*=.038 Performance of the upper limb (transfer phase 0-transfer phase 2)
Massetti et al (2018) [22], Netherlands	22 DMD individuals divided in two groups of 11. Group A started with virtual task; group B started with a real task.	All participants decreased the movement time from the first to the last block of acquisition, more who started with the virtual task. In both virtual and real tasks, motor learning could be inferred by the short-term retention and transfer task (with increasing distance of the target). There was no transference of learning between environments. Only the performance on acquisition phase predicted the degree of learning. MFM^e^ punctuation did not.	Real task in acquisition phase: movement time=746ms. Virtual task in acquisition phase: movement time=1011ms.
Quadrado et al (2017) [23], Brazil	IG=32 individuals with DMD; CG=32 typically developing individuals. 12-32 years old (mean=18 years).	Acquisition phase: for two groups the tendency was late. Absolute timing error was larger in IG group than in the CG. Only absolute timing error in the acquisition phase predicted the amount of learning; age and MFM did not. Transfer phase: for both the CG and IG, completion of real-life tasks did not improve completion of virtual tasks. However, training in the virtual environment did improve real-life task completion.	Absolute timing error IG 1st attempt media (movement time=255 milliseconds) Last attempt (movement time=156 milliseconds) CG (movement time=245 milliseconds) larger than IG. Final acquisition (movement time=156 milliseconds) Variable timing error transfer phase (movement time=369 milliseconds) Final acquisition (movement time=132 milliseconds)
Malheiros et al (2015) [24], Brazil	IG=42 individuals with DMD; CG=42 typically developing individuals. 5 to 18 years old.	Acquisition phase: a significant decrease was found in movement time between the first and last acquisition block, but only for the IG. Movement time: first acquisition=8.4 seconds; last acquisition=5.7 seconds. In the transfer phase movement time increased from retention to transfer: IG=5.7-6.6 seconds; CG 3.3-4.0 seconds.	Movement time during the transfer phase was shorter than during the first acquisition in the IG. Significant effects were found for number of attempts but not for interface type.
De Freitas et al (2019) [17], Brazil	IG=60 male individuals with DMD; CG=60 typically developing male individuals. 9-34 years old. Divided with cross sectional design in three groups of 20 each one.	Acquisition phase: significant effects were found for attempt but not for the device used. Both groups increased the number of balls touched from first to last attempt. IG performance in all interfaces was worse in Touch Screen than Kinect and Leap Motion. CG had better performance for touch screen. Age did not influence the learning effects for the gaming task. Mean score in MFM in standing position and transfers was approximately 15%. Punctuation in Vignos score and MFM in axial and proximal limb motor function predicted the improvement of performance in IG individuals.	Acquisition phase First attempt (M^e^=70), last attempt (M=78), IG (M=57), CG (M=91) Touch Screen: CG: M=105; IG: M=50 Leap Motion: CG: M=86; IG: M=62 Kinect: CG: M=81; IG: M=54 Retention Phase (R) IG in Leap Motion. Last attempt (M=67)–retention (M=81). CG: retention (M=112)–transfer phase 1 (M=76). IG: retention (M=81)–transfer phase 1 (M=59).

^a^DMD: Duchenne muscular dystrophy.

^b^IG: intervention group.

^c^CG: control group.

^d^MFM: Motor Function Measurement.

^e^M: median number of balls collected.

**Table 3:** Summary of measures, devices used, and types of virtual reality game interventions of the included studies.

Author, Country	Measurement instruments	Device/task	Virtual reality game intervention	Duration of study
Correa et al (2009) [18], Brazil	Likert Scale: motivation scale. User measures: ease of use, exercise effect, and satisfaction. Therapeutic measures: practicability of equipment and degree of patient’s motivation.	GenVirtual: a virtual reality–based game that simulates the sounds of musical instruments.	Elbow and wrist extension to play 3-dimensional colored cubes that simulate sounds of musical instruments.	30 minutes of intervention given on a weekly basis with a total of 12 sessions.
Capelini et al (2017) [5], Brazil	Vignos Scale Egen Klassification Scale MFM^a^ in dimension 1 (standing position and transfers), dimension 2 (axial and proximal limb motor function), and dimension 3 (distal limb motor function). Dependent variable: movement time in seconds.	Smartphone: Nokia 500 Game: Marble maze classic (Labyrinth)	Individuals have to direct a virtual ball through a path maze and reach the final target in the shortest time possible. Different paths were used. Three phases were used: (1) acquisition, (2) retention, (3) transfer, which was divided into transfer phase 1, transfer phase 2, and transfer phase 3.	The authors did not describe the duration of the study.
Heuntinck et al (2018) [21], Brazil	Principal measurement was performance of the upper limb. MFM in dimension 3 (distal limb motor function). Kidscreen-52 (quality of life). Muscle strength: hand held Dynamometer Maximum voluntary isometric contractions Vignos Scale	Play station II with Eyetoy using a dynamic arm support. Gainboy (Gravity compensation of 100% in horizontal plane)	Performance of virtual reality games with dynamic arm support (Gainboy). Individuals were trained at home and supervised by a coach.	5 weekly sessions of 15 minutes for a total of 20 weeks.
Massetti et al (2018) [22], Netherlands	MFM Time to perform in milliseconds.	Kinect Sensor MoVer software which allowed the creation of different tasks, during which participants perform functional movements.	An avatar interacts with the object. The object was represented as a red cube, and the participants received visual feedback with the image of their body movement in the virtual environment. Three phases were used: (1) acquisition, (2) retention, (3) transfer, which was divided into transfer phase 1, transfer phase 2, and transfer phase 3.	2 weeks.
Quadrado et al (2017) [23], Brazil	MFM Constant timing error in milliseconds. Absolute timing error in milliseconds. Variable timing error in milliseconds.	A webcam recorded a marker on the table next to the computer keyboard. Software superimposed virtual objects over images of the real world with a webcam. 10 3-dimensional cubes were displayed on a monitor.	The participant had to press the space bar on a keyboard to reach the cube or make a hand gesture with no physical contact at the exact moment the cube turned green. Three phases were used: (1) acquisition, (2) retention, (3) transfer, which was divided into transfer phase 1, transfer phase 2, and transfer phase 3.	The authors did not describe the duration of the study.
Malheiros et al (2015) [24], Brazil	MFM in dimension 1 (standing position and transfers), dimension 2 (axial and proximal limb motor function), and dimension 3 (distal limb motor function). Time to performance Age of individual	﻿Computer maze task. Following a path on the computer screen in the shortest possible time. The authors designed one maze for the acquisition phase and another maze for the transfer phase.	Participants executed a computer maze task; all participants performed the acquisition (20 attempts) and retention (5 attempts) phases, repeating the same maze. A different maze was used to verify transfer performance (5 attempts as well).	The authors did not describe the duration of the study.
De Freitas et al (2019) [17], Brazil	MFM in dimension 1 (standing position and transfers), dimension 2 (axial and proximal limb motor function), and dimension 3 (distal limb motor function). Vignos Scale Age of population	Three interfaces were used: (1) Kinect for Windows, (2) Leap Motion (a virtual interface that required nonphysical contact), (3) a touch screen monitor that required physical contact.	All participants performed tasks using the 3 interfaces. There was an acquisition phase (during which participants practiced the task), a retention phase (1 attempt after 5 minutes), and 2 transfer phases (during which participants were given an attempt to use each device not used in the acquisition phase). Participants had to touch a ball on the screen in and outside the range of movement zone, challenging the limits of individuals.	There were 3 phases of the study for a total of 30 minutes.

^a^MFM: Motor Function Measurement.

## Discussion

Virtual reality technologies have been used in the study of upper limb rehabilitation in recent years and for various conditions such as strokes, cerebral palsy, and neuromuscular diseases. Shin et al [15] found that virtual reality improved treatment outcomes for the distal upper extremity, including motor impairment, hand function, and quality of life in stroke survivors. Jannink et al [13] found that the Eye Toy tool had potential to improve arm function in children with cerebral palsy. De Freitas et al [17] found that those with Duchenne muscular dystrophy benefitted from the use of virtual technologies.

### Methods and Population Classification

The evaluation of study participants’ functionality and overall muscle performance was mostly done using psychosocial variables and scores obtained using the Vignos Scale [17,21]. The Motor Function Measure Scale was also used by the majority of authors chosen for the review [17,21,22,24].

Although those with Duchenne muscular dystrophy can benefit from the use of virtual reality technologies, the technologies in question should be selected carefully and with mind to participants’ unique abilities and needs [17,22]. The studies reviewed agree on the importance of choosing a suitable task for individuals with Duchenne muscular dystrophy due to the generalized weakness that these individuals experience, especially in the upper limbs at the level of the forearm and in the antigravity muscles of the shoulder and elbow in advanced stages [17,23]. For this reason, the pattern of movement required by a task will determine the difficulty of that task [5,17,23]. Many studies found that patients with greater muscle weakness and loss of functionality had lower speeds of movement and worse performance during assigned tasks [5,17].

According to Masseti et al [22], De Freitas et al [17] found that individuals with Duchenne muscular dystrophy exhibited better responses in virtual environments than in the real world or when using touch interfaces. On the other hand, there was no relationship between performance of virtual reality tasks and general functionality (Motor Function Measurement-total), nor between performance of virtual reality tasks and functionality of the hands (Motor Function Measurement-D3) [5,17,23]. However, these results are based on a previous study in which training did not significantly improve performance of the upper limb (performance of upper limb=3.4). This training consisted of playing PlayStation II games for 20 weeks, which challenged participants to move their upper limbs with the help of a dynamic arm support (Gainboy) that compensated for gravity [21].

The studies of Heuntick et al [21] and Massetti et al [22] compared individuals with Duchenne muscular dystrophy in both the experimental and control groups. The other 5 studies included in our review compared a Duchenne muscular dystrophy group with a typical development control group [5,17,18,23,24]. The Duchenne muscular dystrophy groups always recorded a slower result and had lower scores (time to perform) in the acquisition phases [5,18,23,24].

### Metrics and Results

Regarding the metrics used and results obtained, De Freitas et al [17] reported that age did not influence participants’ functionality improvements or their ability to complete virtual tasks, even with the muscle deterioration caused by the progress of the disease.

The authors of the 7 papers we reviewed debated the influence of the Motor Function Measurement variable. De Freitas et al [17] did not find a relation for Motor Function Measurement-D1 in the performance of the task, while Capelini et al [5] did find a connection when using a smartphone. This may be why individuals in the Capelini et al study had a lower mean Motor Function Measurement-D1 score than individuals in the De Freitas et al study (De Freitas et al=12%, Capelini et al=15%) [5,17]. De Freitas et al [17] also reported that participants with more severe motor impairment and fewer motor skills in the proximal musculature (which corresponds to a higher score in Vignos and a lower score in Motor Function Measurement-D2) exhibited a greater capacity to complete homework, and learning was greater. This is, in part, because the score with which the participants started was also lower and, therefore, the range of improvement after training was broader.

Quadrado et al [23] performed a transfer phase with a change in velocity, and only Quadrado et al measured the time of the task. Massetti et al [22] used the variable “time to perform” in the same phases as Malheiros et al [24]. However, Massetti et al used a crossover model with Duchenne muscular dystrophy patients whereas Malheiros et al had an unaffected control group.

In relation to the transfer of tasks, the results of the study by Quadrado et al [23] show that the realization of the task in a virtual environment facilitated the transfer to the real environment, but that the difficulty of the task must be adjusted to be more difficult in the virtual environment in order to facilitate the transfer to the real environment. This may be attributed to the fact that the space-time organization of the virtual task is different from the real one, reducing performance in the virtual environment [22,23]. Massetti et al [22] states that there is no transfer between environments due to the difference in the complexity of tasks. For example, while Quadrado et al [23] required only the movement of the hand to press a key, Massetti et al [22] required the entire upper limb to hit real and virtual cubes. Heuntick et al [21] supported the idea that if the training is transferred from a virtual environment, the effectiveness may be greater than standard exercises with low resistance, which are normally used to work muscles in isolation rather than general movements aimed at functionality.

### Limitations

The variability of the samples in the studies and the differences between the registered variables made it impossible to carry out a meta-analysis to complete the review. The correlation between the type of task assigned to the participants and the participants’ cognitive demand was not measured, nor was the correlation between the type of task and the participants’ quality of movement [5,22,24]. The preparation of the environment or the accuracy of the devices was not specified and may have caused measurement errors [24,25]. The amount of audio-visual stimulus in some cases caused anxiety and did not produce the desired effects in low mobility individuals compared to conventional treatments [18]. The wide range of devices used makes it difficult to compare the results of the studies. The only similar devices were smartphones [5] and computer keyboards [24]; tasks at the motor level were completely different. If we introduce virtual reality as a tool that helps with user motivation and improves the users’ quality of life, these variables should be used in all studies [18,21]. Finally, A great limitation in the use of virtual reality systems is their cost, making it impossible for all users to access.

### Future Directions

The application of virtual reality must go beyond measuring the time for task completion and speed of movement. We must provide studies with specific respiratory and cardiac variables that provide data concerning the metabolic effort during and after exercise and the protection of cardiac activity during exercise. Therapy times and intensities should be defined in individuals with high fatigue and difficulty breathing [26]. Virtual reality can be a great tool for physiotherapy in individuals with Duchenne muscular dystrophy.

Improvement should be measured at different levels in the upper limbs, as well as the proximal level and stabilizing muscles and at the distal and fine motor level [15]. In the Shin et al [15] study, the authors reported benefits at the distal and proximal level when they only expected to find benefits at the distal level, as reported by Capelini et al [5].

### Conclusions

The treatment of neuromuscular diseases has changed in recent years, from palliative symptom management to preventive methods for capacity building. The use of new technologies such as virtual reality may be necessary in the treatment of progressive diseases involving movement difficulties, as virtual reality technologies can provide freedom and improve capacity training results. Given that new technologies are increasingly accessible, rehabilitation and physiotherapy programs more frequently employ virtual reality environments to improve task performance and promote the transfer of this practice to daily life in the real world, which is essential for people with disabilities. Ultimately, virtual reality can be used for Duchenne muscular dystrophy rehabilitation programs to improve individuals’ training performance.
